# Enhancement of salivary human neutrophil peptide 1–3 levels by probiotic supplementation

**DOI:** 10.1186/s12903-015-0003-0

**Published:** 2015-02-10

**Authors:** Onnida Wattanarat, Anupong Makeudom, Thanapat Sastraruji, Supatcharin Piwat, Sukanya Tianviwat, Rawee Teanpaisan, Suttichai Krisanaprakornkit

**Affiliations:** Department of Pediatric Dentistry and Orthodontics, Faculty of Dentistry, Chiang Mai University, Chiang Mai, 50200 Thailand; Department of Medical Technology, Faculty of Associated Medical Sciences, Chiang Mai University, Chiang Mai, 50200 Thailand; Center of Excellence in Oral and Maxillofacial Biology, Department of Oral Biology and Diagnostic Sciences, Faculty of Dentistry, Chiang Mai University, Chiang Mai, 50200 Thailand; Common Oral Diseases and Epidemiology Research Center, Faculty of Dentistry, Prince of Songkla University, Songkhla, 90112 Thailand; Rural Oral Health Center of Southern Thailand, Faculty of Dentistry, Prince of Songkla University, Songkhla, 90112 Thailand; Department of Preventive Dentistry, Faculty of Dentistry, Prince of Songkla University, Songkhla, 90112 Thailand; Department of Stomatology, Faculty of Dentistry, Prince of Songkla University, Songkhla, 90112 Thailand

**Keywords:** Alpha-defensins, Dental caries, Mutans streptococci, Probiotics, Saliva

## Abstract

**Background:**

Probiotic supplementation can reduce mutans streptococci (MS) numbers. One of its proposed mechanisms is immunomodulation. Salivary human neutrophil peptide 1–3 (HNP1-3) levels have previously been demonstrated to be higher in caries-free than in caries-susceptible children, suggesting their preventive role against caries. We aimed to compare salivary HNP1-3 levels between an intervention group with probiotics and a control group.

**Methods:**

A randomized double-blinded clinical trial was conducted. Sixty schoolchildren were equally allocated to either an intervention or control group. The use of a probiotic strain, *Lactobacillus paracasei* SD1, has shown to reduce MS numbers in volunteers. In unstimulated whole saliva, HNP1-3 levels were assayed by ELISA, and MS and lactobacilli counts were assayed by colony counting at baseline (T0) and at 3 (T3), 6 (T6), and 12 months (T12). The International Caries Detection and Assessment system was used to assess caries status.

**Results:**

In the intervention group, salivary HNP1-3 levels were significantly greater than those in the control group at T3 and T6 (*p* < 0.001), whereas MS counts were significantly decreased (*p* < 0.01). In the intervention group, positive and negative correlations were found between HNP1-3 levels and lactobacilli counts and between MS and lactobacilli counts, respectively. However, there was no significant correlation between enhanced HNP1-3 levels and decreased MS numbers. The caries increment for the pit and fissure surface, but not for the smooth surfaces, was significantly decreased in the intervention group compared with the control group (*p* = 0.01).

**Conclusions:**

Probiotics can temporarily enhance salivary HNP1-3 levels; however, their action to reduce new pit and fissure caries probably involves microbial interactions.

**Trial registration:**

TCTR20130904001 (registration date: September 04, 2013).

**Electronic supplementary material:**

The online version of this article (doi:10.1186/s12903-015-0003-0) contains supplementary material, which is available to authorized users.

## Background

Dental caries is one of the most prevalent diseases in both children and adults worldwide [1]. However, the success of all caries preventive programs has been impeded by its multifactorial nature. The disease is a result of demineralization, caused by the interactions of cariogenic bacteria, a diet rich in fermentable carbohydrates, and host components, such as tooth and saliva properties [2]. Although many bacterial species can play a role in the caries process [3], mutans streptococci (MS) have been considered major pathogens associated with early caries development [4]. The main virulence properties of MS are acidogenicity, acid tolerance, biofilm formation and tooth adhesion [5]. Strong association between MS quantities and the pathogenesis of dental caries is demonstrated by several previous studies, reviewed in [6].

Probiotic administration is considered a potential strategy for improving or maintaining oral health. According to the World Health Organization (WHO), probiotics are “live microorganisms which, when administered in adequate amounts, confer a health benefit on the host” [7]. Several mechanisms have been proposed for the probiotic action, including production of antimicrobial substances, competition with pathogens by preventing cellular adhesion and invasion, and modulation of local and systemic immune functions [8-11]. In a recent systematic review [12], several clinical trials have shown the capacity of probiotic supplementation to reduce MS counts, but the decreasing effect is variable and short-lasting. Moreover, only very few studies have so far investigated the quantities of lactobacilli (LB) and the prevention of new caries occurrence by probiotic supplementation.

Although LB, commonly used as probiotics, have been associated with caries progression [13], a recent study has revealed that only a certain species, *i.e.*, *Lactobacillus salivarius*, is more related to caries development by its ability to produce high levels of acids [14]. In contrast to these cariogenic bacteria, *Lactobacillus paracasei* isolated from caries-free subjects possesses an ability to suppress MS growth [15,16]. In this study, *Lactobacillus paracasei* SD1 was introduced as a probiotic strain and used in the oral cavity because of its several previously-demonstrated properties, including inhibition of MS growth, less acid production than other LB, and good adherence to oral epithelial cells [17].

Human neutrophil peptides 1–3 (HNP1-3) are small cationic antimicrobial peptides that provide the first line of host defense against a broad spectrum of microorganisms [18]. HNP1-3 are expressed in ductal epithelial cells of submandibular salivary glands and secreted into saliva [19]. They are also produced by neutrophils and released into gingival crevicular fluid [20]. The preventive role of HNP1-3 against dental caries has been suggested by the significantly higher salivary HNP1-3 levels in caries-free children than in those experiencing caries demonstrated by Tao et al. [19]. Since one of the probiotic mechanisms has been proposed to be involved with host immune regulation, we, therefore, hypothesized that probiotic supplementation might help prevent dental caries by augmentation of local host immunity via enhanced production of salivary HNP1-3. The aims of this study were to examine the effects of probiotic intervention on salivary HNP1-3 levels, MS counts, and LB counts, and to determine the correlations between these host and microbial parameters. Furthermore, new occurrences of carious lesions for the second permanent molars during a 12-month clinical trial were assessed and compared between the control and intervention groups.

## Methods

### Overview of study design

**Figure 1:**
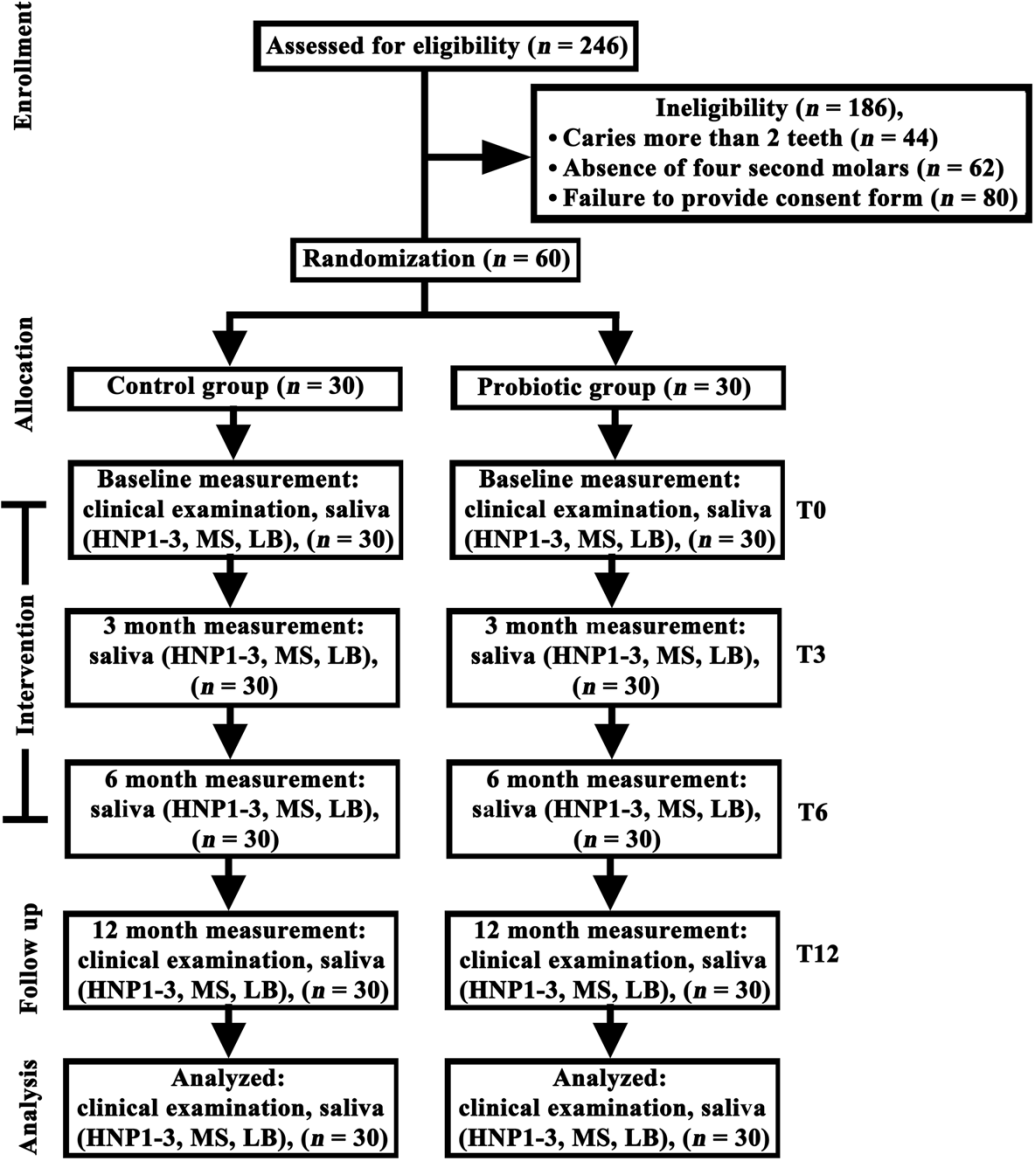
**A consort flow chart showing the number of participants in the control and probiotic groups at the beginning and completion of the trial.** Out of 246 children enrolled in this trial, 60 participants remained eligible due to the inclusion and exclusion criteria. Sixty participants were equally allocated to either the control or the probiotic group by simple randomization described in the Methods. Participants received milk powder with or without probiotics during the first six-month intervention, and saliva samples were collected for analyses of HNP1-3 levels, MS and LB counts at four different time points: T0, T3, T6 and T12. Oral examination for evaluation of ICDAS scores was performed at T0 and T12.

This trial was designed as a randomized, double-blinded and placebo-controlled method with two parallel groups. The study protocol was approved by the Human Experimentation Committees of the Faculties of Dentistry, Chiang Mai University and Prince of Songkla University, Thailand. Informed consent was obtained from the parents or guardians of each participant before the commencement of this study. Sixty eligible and healthy participants out of 246 children were allocated equally to either of the two groups, the control and probiotic groups, using a simple randomization procedure by means of drawing lots (Figure 1). The inclusion and exclusion criteria are identified in Participants and in Figure 1. For the double-blinded method, the code was kept by an independent monitor. This code was not unveiled until all data had been analyzed. None of the researchers, the clinicians, the participants, the teachers, or the statistician knew whether the children received control or intervention milk throughout the entire course of this study. The trial was registered at the http://www.clinicaltrials.in.th/, one of the primary WHO Registry Networks, Clinical Trials identifier TCTR20130904001. The whole experimental period lasted for 12 months, and the outcome measurement of this study consisted of four parameters: salivary HNP1-3, MS and LB levels at four different time points: T0 (baseline), T3 (three months of intervention), T6 (six months of intervention) and T12 (six months after cessation of the intervention), and the presence of carious or demineralized lesions at T0 and T12 (Figure 1). The *priori* sample size calculation was performed with a focus on mean differences of MS counts between two independent groups using G Power software [21], with the effect size equal to 0.8 at 5% statistical significance level and 90% power of test. The calculation yielded no fewer than 28 participants in each group.

### Preparation of milk powder supplemented with probiotics

The probiotic strain, *Lactobacillus paracasei* SD1, was isolated from caries-free volunteers and has been previously shown to exert the maximal inhibitory effect on *Streptococcus mutans in vitro* [17]. The bacterial strain was identified by PCR-RFLP of the 16S ribosomal RNA gene and sodium dodecyl-polyacrylamide gel electrophoresis [22]. The probiotic intervention was manufactured in a form of milk powder by the spray drying technique. The culture conditions and the spray drying technique were performed as previously described [23]. Briefly, a culture of *Lactobacillus paracasei* SD1 was inoculated in a mixture of 1% probiotics and a 3-liter quantity of heat-treated (50°C for 30 min) and 20% reconstituted skimmed milk, and then spray dried with a spray dryer (model B191 Buchi mini spray dryer; Flawil, Switzerland). The final product was yellowish white powder with moisture contents of 3.44 ± 0.85% and viable counts of 7.5 ± 0.20x10^8^ cfu/g, which was stored at 4°C. The viability of *Lactobacillus paracasei* SD1 in the inoculated milk powder was previously assessed in a six-month study by examining its growth in triplicate Man Rogosa and Sharpe (MRS) pour plates under anaerobic incubation for three days at 37°C [23]. It was demonstrated that the maximum survival rate of *Lactobacillus paracasei* SD1 in the skimmed milk powder was 99% if the milk powder was stored at 4°C for six months [23]. However, the survival rate declined sharply if the milk powder was stored at 25°C [23]. The milk powder in general appearance for both probiotic and control groups looked identical, except for the absence of the probiotic strain in the control group, and was packaged in the same clear plastic bags marked with a name of each participant. The packaging and delivering steps were performed by a research assistant who was blinded from every step of sampling and analyzing.

### Participants

The inclusion criteria were: (i) good oral health with caries in two or fewer teeth (ii) presence of four second molars in the mouth (iii) absence of untreated active deep carious lesions (iv) absence of periodontitis (v) non-smoking and (vi) daily tooth brushing habit using fluoride toothpaste. The exclusion criteria were teenagers with systemic diseases, receiving systemic antibiotics within six weeks, routine consumption of probiotics or xylitol, allergy to cows’ milk, lactose intolerance and severe food allergy. Prior to the commencement of the clinical trial, all participants were informed not to consume any products containing probiotics, such as yoghurt, drinking yoghurt, cheese, etc., throughout the entire duration of this trial. The participants in both groups were instructed to mix 5 g of milk powder in 50 ml water and drink once daily, except on the days of saliva collection, for six months under the supervision of instructors. On the holidays or the weekends, all children were informed to drink their milk at home and to return the empty plastic bags. The compliance was checked by their teachers who filled in a logbook every day with information on school attendance of children and on whether or not the children had been drinking the milk. It was apparent that no children participating in this study were absent from their classes due to sick leave during the first six-month intervention. All participants were asked to immediately report any side effects.

### Analyses of salivary HNP1-3 levels

A 2-ml quantity of unstimulated whole saliva was collected and equally divided in two aliquots, one for analysis of HNP1-3 levels and the other for analysis of microbiological levels. The first aliquot was added with Nonidet P-40 (Sigma-Aldrich, St. Louis, MO) to the final concentration of 0.1% (v/v), centrifuged at 15,000 rpm at 4°C for 10 min [19], and the cleared supernatant was collected and stored frozen for further analysis of HNP1-3 levels by an HNP1-3 ELISA kit (HyCult Biotechnology, Uden, the Netherlands) according to the manufacturer’s instruction. In brief, diluted saliva samples (1:200) were applied to a pre-coated plate in triplicate with the specific primary antibody to HNP1-3, and incubated for 1 h at room temperature. For a negative control, dilution buffer without addition of the saliva samples was added to the pre-coated plate. After washing four times with washing buffer, each well was incubated with the biotinylated tracer antibody for 1 h at room temperature. Then, the streptavidin-peroxidase conjugate was added and incubated for 1 h. After that, 3,3′,5,5′-tetramethylbenzidine, a chromogenic substrate, was added for 20 min, and the reaction was then stopped by the addition of 2% oxalic acid. The concentrations of HNP1-3 in saliva samples were calculated from a standard curve established by various known concentrations of an HNP1-3 standard, used as the ELISA system control. The concentrations of salivary HNP1-3 were then normalized by their total protein content using a BCA protein assay (Pierce Inc., Rockford, IL) according to the manufacturer’s instruction.

### Microbiological assays

The quantities of salivary MS and LB were evaluated by a typical colony counting method at T0, T3, T6 and T12. The second aliquot of saliva samples was ten-fold diluted from 1:10 to 1:10,000. Each dilution (10 μl) was dropped onto the selective agar plates, Mitis Salivarius Bacitracin (MSB) agar for MS (Difco Laboratories, Detroit, MI) and MRS agar for LB (Difco Laboratories). The conditions for incubation were anaerobic, 10% H_2_, 10% CO_2_ and 80% N_2_ at 37°C for 48 h. The colony counting was performed under a microscope in duplicate.

### International Caries Detection and Assessment System (ICDAS)

At the beginning and the end of this study (T0 and T12), all participants were examined for oral health and dental caries status by two experienced dentists (S.P. and S.T.) with the Cohen’s kappa values 0.85 and 0.82 for the intra- and inter-examiner calibrations, respectively. The examination was performed by using a mouth mirror and an air syringe under an operating light. The caries data for each tooth surface, including occlusal (pit and fissure) and smooth surface (buccal, lingual, mesial and distal) caries, were recorded according to the criteria of the International Caries Detection and Assessment System (ICDAS) [24]. The ICDAS codes consist of: 0 = sound, 1 = first visual change in enamel, 2 = distinct visual change in enamel, 3 = localized enamel breakdown, 4 = underlying dentin shadow, 5 = distinct cavity with visible dentin, and 6 = extensive cavity within visible dentin. Caries risk assessment in this study was determined by the modified criteria previously described by Nase and co-workers [25], which include both clinical caries status and MS quantities as follows. The “high risk” participants were defined as having both an ICDAS score >0 and MS levels ≥10^5^ cfu/ml, whereas the “moderate risk” ones had either an ICDAS score >0 or MS levels ≥10^5^ cfu/ml and the “low risk” ones had an ICDAS score =0 and MS levels <10^5^ cfu/ml.

### Statistical analyses

The Mann–Whitney *U* test was used to analyze inter-group differences in salivary HNP1-3 levels. Differences in salivary HNP1-3 levels among four different periods in each group were analyzed by the Kruskal-Wallis test, followed by the Mann–Whitney *U* test. The number of colony counts for MS and that for LB were presented as log cfu/ml and analyzed by the independent sample *t*-test. Correlations between each pair of the data from HNP1-3 levels, MS and LB counts in each group were tested by the Pearson Correlation test. Regression analysis was performed for the significant correlation data. An odds ratio (OR) for caries increment was calculated by Crosstabulation. The software package used was the Statistical Package for Social Sciences (SPSS version 17.0 Inc., Chicago, IL), and the differences were considered significant when *p*-values were less than 0.05. All of these statistical methods were performed by a biostatistician (T.S.).

## Results

### Demographic data

A total of 60 participants (age ranges 13–15 years; *n* = 26 for males and 34 for females) completed this 12-month clinical trial (Figure 1). No adverse side effects from probiotics or milk powder intake in this cohort were reported. The compliance for daily consumption of milk powder in both groups was carefully monitored under the supervision of schoolteachers and well controlled throughout the first six-month intervention period. The assessment for caries risk revealed that the percentages of participants classified in the “high risk”, the “moderate risk” and the “low risk” groups were 50, 31.7 and 18.3, respectively. Although the randomized procedure was performed to allocate all 60 eligible participants to the control or probiotic group, it was coincident that the percentages of high, moderate and low caries risk in the control versus the probiotic group were almost equal as follows: 50.0 versus 50.0, 33.3 versus 30.0, and 16.7 versus 20.0, respectively.

### Raised salivary HNP1-3 levels in the probiotic group

**Figure 2:**
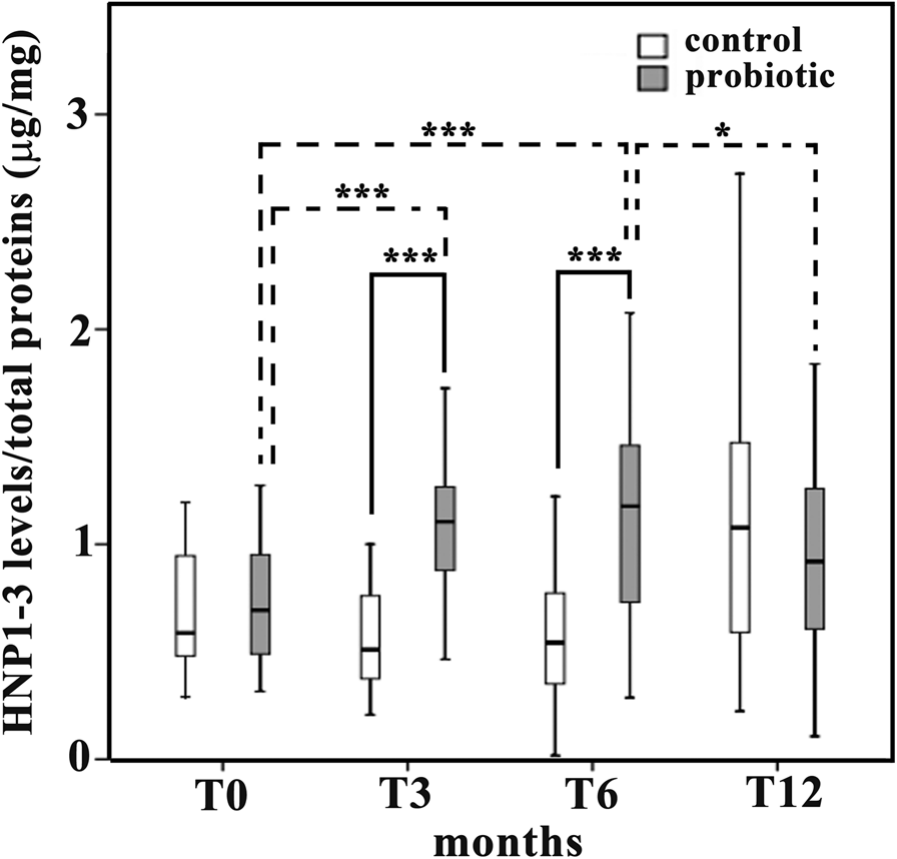
**Significant but transient increase in salivary human neutrophil peptide (HNP) 1–3 levels by probiotic supplementation.** The *y*-axis of box plot graph demonstrates salivary HNP1-3 levels normalized by total protein concentration in the unit of μg/mg for the control (empty boxes) and the probiotic (gray boxes) groups from four different periods of saliva collection: T0, T3, T6 and T12. * = *p* < 0.05; *** = *p* < 0.001. The solid lines show the significant differences between two groups, while the dotted lines represent the significant differences within the probiotic group.

The median salivary HNP1-3 levels, expressed in the unit of μg/mg of total proteins, in the probiotic group were significantly greater than those in the control group at T3 (1.0250 versus 0.5145 μg/mg; *p* < 0.001) and T6 (1.1470 versus 0.5415 μg/mg; *p* < 0.001), but not at T0 or T12 (Figure 2). With respect to the kinetics of salivary HNP1-3 levels in the probiotic group during the 12-month period of this clinical trial, it was demonstrated that the HNP1-3 levels were temporarily elevated during the six-month intervention period (Figure 2). In other words, these levels were significantly increased at T3 and T6 (*p* < 0.001) and gradually declined at T12. However, no significant changes in salivary HNP1-3 levels in the control group at four time points (T0, T3, T6 and T12) were observed (Figure 2).

### Decreased MS counts as opposed to increased LB counts by probiotic intervention

**Figure 3:**
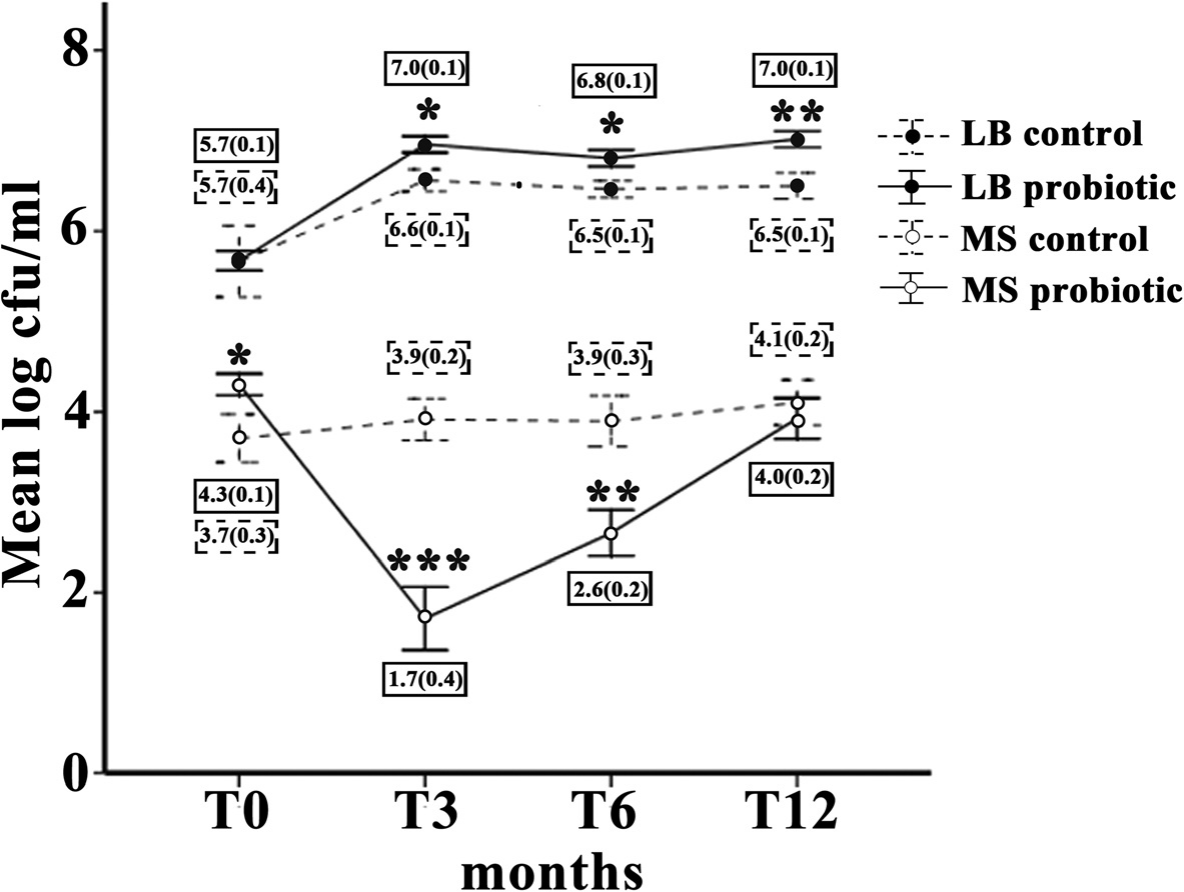
**Significant but transient reduction in salivary mutans streptococci (MS) counts by probiotic intervention.** The linear graph illustrates mean log counts of MS (open circles) and those of lactobacilli (LB; black circles) in the unit of cfu/ml (*y*-axis) in both probiotic (solid lines) and control (dotted lines) groups from four different periods of saliva collection (*x*-axis): T0, T3, T6 and T12. * = *p* < 0.05; ** = *p* < 0.01; *** = *p* < 0.001. Error bars represent standard errors (SE). The values of mean log MS and LB counts and (SE) are shown in the solid boxes for the probiotic group and in the dotted boxes for the control group in each period of saliva collection.

The mean counts and standard errors for salivary MS and LB (log cfu/ml) at T0, T3, T6 and T12 in the control and probiotic groups are demonstrated in a linear graph (Figure 3). At baseline (T0), it was demonstrated that the mean salivary LB count in the probiotic group (a solid line) was not significantly different from that in the control group (a dotted line), while the mean salivary MS count in the probiotic group was significantly higher than that in the control group (*p* < 0.05; Figure 3). However, at T3 and T6, a significant reduction in MS counts in the probiotic group was clearly evident when compared with those in the control group (T3 = *p* < 0.001 and T6 = *p* < 0.01), whereas a significant increase in LB counts was instead observed (*p* < 0.05; Figure 3). In contrast to the probiotic group, no significant changes in the mean counts for salivary MS and LB were found in the control group (dotted lines in Figure 3). At T12, the mean count for salivary MS in the probiotic group was increased and returned almost to the baseline, while the mean count for salivary LB in the probiotic group was still significantly higher than that in the control group (*p* < 0.01; Figure 3), suggesting the ability of LB to be retained in the oral cavity despite cessation of the probiotic intervention for six months.

### Significant reduction in the caries increment for the pit and fissure surface in the probiotic group

**Figure 4:**
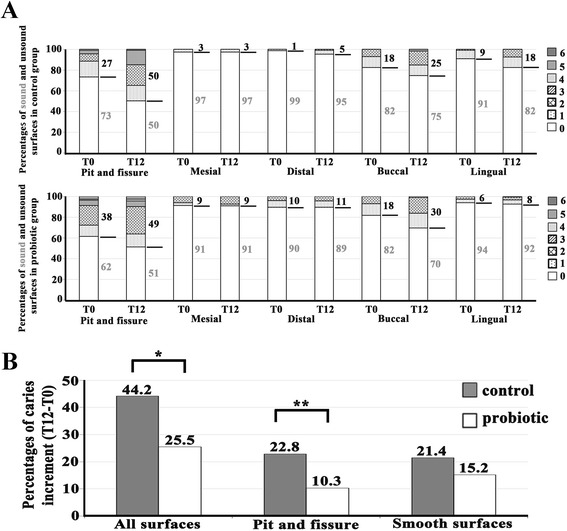
**Significant decrease in the percentage of caries increment for the pit and fissure surface, but not for the smooth surfaces, by probiotic intervention. (A)** The 100% stacked bars demonstrate the percentages of sound (white area; ICDAS score =0) and unsound (filled areas; ICDAS scores =1–6) surfaces from all five tooth surfaces, including pit and fissure, mesial, distal, buccal, and lingual, in the control (upper panel) and the probiotic (lower panel) groups. Note higher percentages of unsound surfaces in the pit and fissure surface than those in the other four surfaces. **(B)** The bar graph reveals significant reduction in the percentages of caries increment in the probiotic group for a combination of all five tooth surfaces and for the pit and fissure surface alone, but not for the combined four smooth surfaces, including mesial, distal, buccal and lingual surfaces. * = *p* < 0.05; ** = *p =* 0.01.

The percentages of sound (ICDAS score =0) and unsound surfaces (ICDAS scores =1–6) from all five tooth surfaces, including pit and fissure, mesial, distal, buccal, and lingual, at T0 and T12 in both control and probiotic groups are illustrated as stacked bars to a total of 100% (Figure 4A). It was apparent that the percentages of carious lesions in the second permanent molars in both control and probiotic groups increased in almost all tooth surfaces during this 12-month study and that the pit and fissure caries in both groups was more prevalent in our cohort than was caries in the other four smooth surfaces, including mesial, distal, buccal and lingual (Figure 4A). Sums of the percentages of caries increment for all five tooth surfaces, including the pit and fissure surface and four smooth surfaces, and for four smooth surfaces in the probiotic group versus the control group were 25.5 versus 44.2 and 15.2 versus 21.4, respectively (Figure 4B). The percentages of caries increment only in the pit and fissure surface were 10.3 versus 22.8 (Figure 4B). Interestingly, the percentage of new caries occurrence for all five tooth surfaces (OR = 1.605; 95% CI 1.007–2.557; *p* = 0.045) and for the pit and fissure surface (OR = 2.582; 95% CI 1.235–5.400; *p* = 0.01) was significantly lower in the probiotic group than that in the control group, whereas there was no significant difference in new caries occurrence between two groups for the four smooth surfaces (OR = 1.506; 95% CI 0.726–3.126; *p* = 0.269).

### Lack of correlation between increased levels of HNP1-3 and decreased numbers of MS

**Figure 5:**
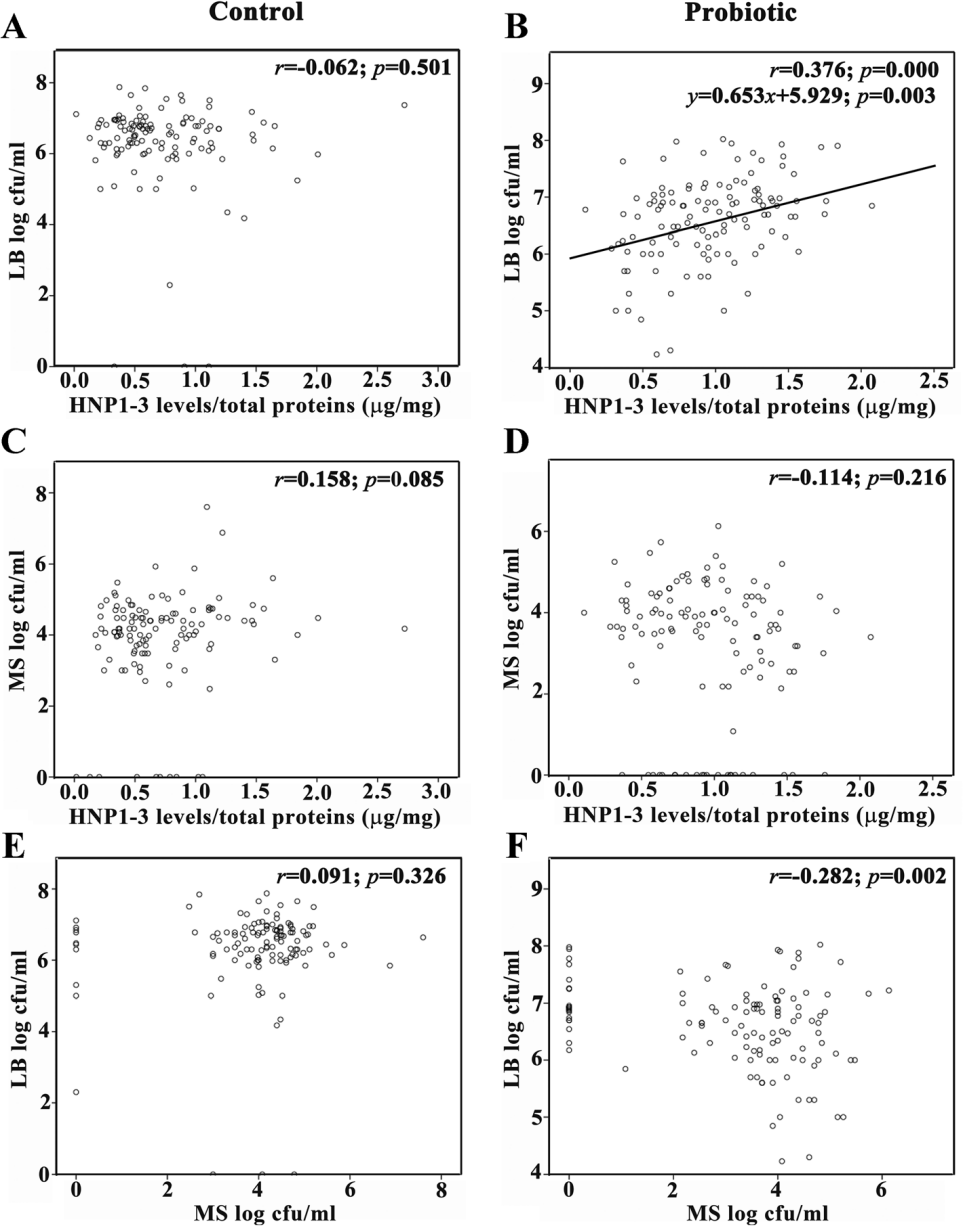
**Positive and negative correlations between salivary human neutrophil peptide (HNP) 1–3 levels and log counts of lactobacilli (LB) and between log counts of mutans streptococci (MS) and those of LB, respectively, in the probiotic group.** The correlation coefficients (*r*) and the significance levels (*p*-values) between each pair of three parameters, including HNP1-3 levels in μg/mg, LB counts in log cfu/ml and MS counts in log cfu/ml were determined and illustrated for both control **(A,** **C and** **E)** and probiotic **(B** **, D and** **F)** groups. The regression analysis could be determined only for the significant correlation between increased HNP1-3 levels and increased LB counts in B, and a regression line was drawn with the equation: *y* = 0.653*x* + 5.929; *p* = 0.003.

Correlations between each pair of salivary HNP1-3 levels in μg/mg of total protein, MS counts and LB counts in log cfu/ml in both control and probiotic groups were determined by the Pearson Correlation test (see Additional file 1 for the data of each parameter), and it was revealed that significant correlations between these parameters were only found in the probiotic group, but not in the control group (Figure 5). Particularly, salivary HNP1-3 levels were positively correlated with LB counts (*r* = 0.376; *p* < 0.001; Figure 5B) with the equation from the regression analysis: *y* = 0.653*x* + 5.929, where *y* is the log LB count and *x* is the HNP1-3 level, whereas LB counts were inversely associated with MS counts (*r* = −0.282; *p* = 0.002; Figure 5F). Nevertheless, no significant correlation was noted between salivary HNP1-3 levels and MS counts in the probiotic group (Figure 5D), suggesting that enhanced salivary HNP1-3 levels by the probiotic intervention are not associated with decreased MS quantities.

## Discussion

The main findings from this study include (a) a significant but temporary increase in salivary HNP1-3 levels at T3 and T6 in the probiotic group; (b) a significant increase in LB counts as opposed to a transient decrease in MS counts during probiotic intervention; (c) positive and negative correlations found between HNP1-3 levels and LB counts and between LB counts and MS counts, respectively, in the probiotic group; and (d) a significant reduction in caries increment for the pit and fissure surface, not for the smooth surfaces. Among these four major findings, the significant increase in salivary HNP1-3 levels by probiotic supplementation during the six-month intervention period is of great interest since it is probable that *Lactobacillus paracasei* SD1 may exert an immunostimulatory effect by enhanced production of innate immune effectors, especially HNP1-3, from ductal epithelial cells of submandibular salivary glands [19], which are then secreted into the saliva. The possible immunostimulatory effect of *Lactobacillus paracasei* SD1 warrants further investigations. In addition, the innate immune activation by *Lactobacillus paracasei* SD1 is corroborated with the significant correlation between elevated salivary HNP1-3 levels and increased LB counts seen in Figure 5B. Activation of host immunity, particularly increased production of secretory immunoglobulin A (sIgA), has been previously shown to be one of the principal mechanisms for probiotics in the gastrointestinal tracts [26-28] and in the oral cavity [29-31]. In this study, the rationale to investigate only the salivary HNP1-3 levels during probiotic intervention was based on the observation from a single previous study that demonstrated significantly higher HNP1-3 levels in the saliva samples from caries-free children than those from caries-susceptible children [19]. However, it is necessary to further determine the salivary levels of other immune-related effector molecules, especially other antimicrobial peptides besides HNP1-3, in a future study. It is noted that the levels of salivary HNP1-3 detected in our cohort were comparable to those measured by the same ELISA method performed by Tao and co-workers [19]. Interestingly, the HNP1-3 levels were temporarily elevated during the six-month intervention period and gradually declined to the baseline levels after cessation of probiotic intake. The temporary induction of HNP1-3 levels was in line with the transient induction of sIgA levels in saliva by probiotic intake [30], indicating that daily consumption of probiotics seems to be required for a sustainable increase in host immune responses.

Nevertheless, the raised salivary HNP1-3 levels did not significantly correlate with the decreased MS counts, but the LB counts were instead correlated inversely with the MS counts, suggesting that the mechanism for probiotic action in the oral cavity may probably involve the competition and/or the interaction between two distinct microorganisms as previously shown by Teanpaisan and Piwat [22] rather than enhanced production of HNP1-3. It is worthwhile to note that the inverse correlation between MS and LB counts was weak, so this suggests that other factors that were not tested in this study, such as different MS strains recovered from different individuals [32], may play a role in the regulation of MS numbers by LB. In contrast to no significant change in MS counts in the control group, it was evident that the MS counts were transiently decreased in the probiotic group at T3 and T6, consistent with the finding from a previous study [33]. This, again, confirms the short-lasting effect of probiotics against MS numbers, similar to the transient increasing effect on HNP1-3 levels. It is interesting to note a prolonged increase in LB counts irrespective of the cessation of probiotic intervention, which may be explained by the ability of *Lactobacillus paracasei* SD1 to be retained in the oral cavity, as previously reported [22] and by the counting method for total LB on MRS agar in this study, rather than the specific quantification of *Lactobacillus paracasei* SD1. Consequently, it is possible that the increased MS counts back to the baseline levels at 12 months were attributable to the absence of *Lactobacillus paracasei* SD1, although the total LB counts were still high. In this study, we did not examine the morphology of LB recovered from saliva samples in great detail with Gram stain, although some colonies of LB on MRS plates were randomly selected and checked for the presence of *Lactobacillus paracasei* SD1 by a qualitative assay like Arbitrarily Primed-Polymerase Chain Reaction fingerprints [22]. Therefore, the inability to confirm the morphology of LB and to quantitatively compare the levels of *Lactobacillus paracasei* SD1 between the control and probiotic groups at each time point is a limitation of this study and this inability may preclude the possibility of *Lactobacillus paracasei* SD1 retention in the oral cavity.

In addition to the laboratory results regarding HNP1-3 levels and microbial counts in saliva, we extended our investigation into the clinical findings relating to caries increment in our cohort. The significant reduction by approximately 2.6 fold for caries increment within 12 months was observed for the pit and fissure caries in the probiotic group when compared with the control group (Figure 4B), suggesting that probiotic intervention in milk powder helps reduce the occurrence of new carious lesions for the pit and fissure surface. In addition to considering the pit and fissure surface alone, the sum of the percentages for caries increment in all five tooth surfaces was also significantly reduced in the probiotic group (Figure 4B). These results differ from the findings from two previous studies, which demonstrated a low odds ratio for caries prevention on occlusal surfaces from milk containing *Lactobacillus rhamnosus* GG in preschool children [25] and lack of significant difference in new caries occurrence between the probiotic and control groups [34]. A possible explanation for this discrepancy is that the participants in each of those studies were categorized as low caries risk, whereas half of the participants in our study were categorized as high caries risk, according to the modified criteria for caries assessment mentioned in the Methods. Therefore, as suggested by the results of those studies and ours, probiotic intervention may be beneficial to reduce the new occurrence of pit and fissure caries in children with high caries risk.

In this study, it was demonstrated that the probiotic intervention did not reduce new caries occurrence on the smooth surfaces. This may be explained by the fact that pit and fissure caries is more associated with MS quantities than is smooth surface caries, consistent with the critical role of MS in pit and fissure caries development [35]. Moreover, it was apparent that the effect of probiotics on smooth surface caries varied among different smooth surfaces (Figure 4A). Particularly, the probiotics appeared to have a caries-reducing effect on the distal and lingual surfaces, but not on the mesial and buccal surfaces. Nevertheless, such mixed effects did not reach a statistically significant level when sum of the percentages for caries increment in four smooth surfaces was determined (Figure 4B). Some factors that can influence distinct outcomes between occlusal and smooth surface caries and then preclude statistical verification include the location of second permanent molars (whether in maxillae or in mandibles), the sample size, and the 12-month duration of this study. Therefore, it is suggested that a larger study with a longer follow-up duration and with special attention to the tooth location be further conducted to more precisely verify all of the statistically significant and non-significant findings and to define the extent and mechanism behind any observable beneficial effects from probiotic intervention.

Utilizing non-sweetened milk powder as a suitable vehicle to introduce the probiotics in this study provided several health benefits for the participants, including provision of more nutrients, good compliance, and additional caries prevention from some constituents in milk powder, such as casein, calcium and phosphorous [36]. Moreover, among several different LB species that have been reported as probiotics, it has been previously shown that *Lactobacillus paracasei* exerted a maximum inhibitory effect on MS [15,16], possibly by its ability to produce paracasin SD1 that functions against MS in the oral cavity [37]. Furthermore, compared with various probiotic strains used in other studies, predominantly isolated from the gastrointestinal tract [33,34,38,39], *Lactobacillus paracasei* SD1 used in our study was isolated from the oral cavity of caries-free volunteers. However, this probiotic strain could only exert a temporary effect on MS reduction and HNP1-3 induction despite a prolonged increase in LB counts in the saliva samples. This may be because the colony counting method on MRS agar cannot differentiate our probiotic strain from other LB strains that already reside in the participants’ mouths. Otherwise, it is probable that our probiotic strain can possibly increase the number of other LB strains, a possibility that warrants further investigations.

Although cariogenic progression varies between different individuals and sometimes lasts longer than a year, the 12-month randomized and double-blinded clinical trial was designed in order to control different inherent factors between the control and probiotic groups. Additionally, the ICDAS was chosen as a tool to assess carious lesions in this short period of study because it is sensitive enough to detect an early carious lesion [40]. Further studies into the underlying mechanisms of increased HNP1-3 production by our probiotics in different oral cell types are currently being investigated, and additional studies into the activation of other oral immune effectors classified in acquired and innate immunity are essential to verify the real potential benefits and applications of probiotics for good oral health, especially for caries prevention. Several confounding factors that were beyond our control in each individual, including variations in the baseline levels of salivary HNP1-3, in the different quantities and strains of MS, in the quantities of fermentable carbohydrate consumption, and in the quality of oral hygiene care, are noted and may affect the outcomes of this study.

## Conclusions

Probiotic supplementation with *Lactobacillus paracasei* SD1 can temporarily enhance salivary HNP1-3 levels and decrease the numbers of MS. The significant correlation, found between increased LB counts and decreased MS counts, suggests that probiotic action may involve the competition or interaction between two distinct microorganisms. In this study, the increment of pit and fissure caries, but not of smooth surface caries, was diminished by probiotic supplementation in the form of milk powder.

### Available supporting data

The data of mutans streptococci (MS) and lactobacilli (LB) counts and human neutrophil peptide (HNP) 1–3 levels at T0, T3, T6 and T12 are provided in the Additional file 1.xls on BMC Oral Health website.

## Additional file

Additional file 1:
**Data of mutans streptococci (MS) and lactobacilli (LB) counts and human neutrophil peptide (HNP) 1-3 levels at T0, T3, T6 and T12.**

## Abbreviations

BCA: Bicinchoninic acid assay
cfu: Colony forming unit
ELISA: Enzyme-linked immunosorbent assay
HNP: Human neutrophil peptide
ICDAS: International caries detection and assessment system
LB: Lactobacilli
MRS: Man Rogosa and Sharpe
MS: Mutans streptococci
MSB: Mitis Salivarius Bacitracin
OR: Odds ratio
PCR-RFLP: Polymerase chain reaction-restriction fragment length polymorphism
RNA: Ribonucleic acid
